# Thermal History-Dependent Deformation of Polycarbonate: Experimental and Modeling Insights

**DOI:** 10.3390/polym17152096

**Published:** 2025-07-30

**Authors:** Maoyuan Li, Haitao Wang, Guancheng Shen, Tianlun Huang, Yun Zhang

**Affiliations:** 1 School of Aerospace Engineering, Xiamen University, Xiamen 361005, China; limaoyuan@xmu.edu.cn; 2 State Key Laboratory of Materials Processing and Die & Mold Technology, School of Materials Science and Engineering, Huazhong University of Science and Technology, Wuhan 430074, Chinamarblezy@hust.edu.cn (Y.Z.); 3 Xi’an Modern Chemistry Research Institute, Xi’an 710065, China; 4 Shenzhen Institutes of Advanced Technology, Chinese Academy of Sciences, Shenzhen 518055, China

**Keywords:** polycarbonate, constitutive model, deformation behavior, thermal history

## Abstract

The deformation behavior of polymers is influenced not only by service conditions such as temperature and the strain rate but also significantly by the formation process. However, existing simulation frameworks typically treat injection molding and the in-service mechanical response separately, making it difficult to capture the impact of the thermal history on large deformation behavior. In this study, the deformation behavior of injection-molded polycarbonate (PC) was investigated by accounting for its thermal history during formation, achieved through combined experimental characterization and constitutive modeling. PC specimens were prepared via injection molding followed by annealing at different molding/annealing temperatures and durations. Uniaxial tensile tests were conducted using a Zwick universal testing machine at strain rates of 10^−3^–10^−1^ s^−1^ and temperatures ranging from 293 K to 353 K to obtain stress–strain curves. The effects of the strain rate, testing temperature, and annealing conditions were thoroughly examined. Building upon a previously proposed phenomenological model, a new constitutive framework incorporating thermal history effects during formation was developed to characterize the large deformation behavior of PC. This model was implemented in ABAQUS/Explicit using a user-defined material subroutine. Predicted stress–strain curves exhibit excellent agreement with the experimental data, accurately reproducing elastic behavior, yield phenomena, and strain-softening and strain-hardening stages.

## 1. Introduction

Due to its low density, high transparency, and excellent impact strength [1,2,3,4], polycarbonate (PC) has been widely used in engineering applications, such as safety devices and aerospace components [5]. As noted by Olson et al. [6], the mechanical behavior of polymers is closely linked to their microstructure, which is heavily influenced by the formation process. This microstructural evolution governs key mechanical responses such as yielding, strain softening, and strain hardening [7]. For example, annealed PC specimens have been shown to exhibit higher yield stress and more pronounced strain softening, highlighting the significant effect of the annealing history on mechanical properties [8]. A comprehensive understanding of PC’s deformation behavior under different thermal histories is therefore essential for optimizing mechanical performance in manufacturing [9,10].

Over the past few decades, extensive experimental studies have investigated the influence of formation conditions on the mechanical behavior of polymeric materials. Masato et al. [11] investigated the effects of the processing-induced thermal history on polycarbonate mechanical properties and found that the thermal history has a significant effect at low strain rates, while its influence is negligible in the dynamic range. Govaert, Engels, and others [8,12,13] reported that both the annealing temperature and annealing time positively correlate with yield stress in PC specimens. Similarly, higher mold temperatures during injection molding were found to increase yield stress, attributed to reduced cooling rates and consequent microstructural changes [11]. More recently, Xu and Dar et al. [14,15,16] conducted tensile experiments on PC and observed consistent trends. They also found that fracture strain decreases with an increasing testing temperature, while yield stress increases with a higher back pressure, further confirming that formation conditions significantly affect the deformation behavior of polymers. Meanwhile, Song et al. [17,18] conducted a thorough investigation of temperature rises in polycarbonate and found that experiments at higher strain rates and lower temperatures produce a greater temperature rise because of the higher yield stress.

Based on extensive experimental studies, numerous constitutive models have been developed to characterize the relationship between the formation process and the deformation behavior of polymers. Govaert et al. [8,12,13] made significant contributions toward integrating injection molding simulations with service performance predictions. In their work, yield stress–time curves at various annealing temperatures were unified into a master curve using time–temperature equivalence. These curves were then applied to injection molding simulations, enabling the prediction of yield stress under different mold temperature conditions by integrating the cooling temperature field. This approach was subsequently embedded into a constitutive model to capture the effects of the thermal history during formation on mechanical behavior [19]. Building on this foundation, Xu et al. [15] employed a Johnson–Cook model incorporating the thermal history during formation to accurately predict the yield stress and fracture behavior of PC specimens. Their results demonstrated that yield stress increased with mold temperature, and Izod impact tests revealed that higher mold temperatures require greater impact energy to initiate plastic deformation and fracture. These studies collectively highlight that both service conditions (e.g., temperature and strain rate) and the formation process significantly influence the mechanical response of polymers. Incorporating the thermal history during formation into constitutive models enhances their predictive accuracy. However, despite progress in experimental and theoretical modeling, existing service behavior simulations often remain disconnected from injection molding simulations and fail to fully account for the formation process’s impact.

Therefore, developing an integrated simulation framework that links injection molding and in-service mechanical behavior is of great importance.

In this study, the deformation behavior of PC specimens produced under varying mold temperatures and annealing conditions was experimentally investigated across strain rates ranging from 10^−3^ to 10^−1^ s^−1^ and temperatures from 293 to 353 K. To capture the influence of the thermal history during injection molding, a new constitutive model was developed based on a phenomenological framework proposed in our previous works [20,21,22]. The formation thermal history was obtained through injection molding simulation, enabling the accurate prediction of the yield stress during the molding process. This model was implemented in ABAQUS/Explicit 6.10 via a user-defined material subroutine (VUMAT), allowing the precise simulation of PC’s deformation behavior under different thermal histories. This approach achieves an integrated simulation of the injection molding process and subsequent mechanical response.

## 2. Materials and Methods

### 2.1. Materials and Specimen Preparation

**Figure 1:**
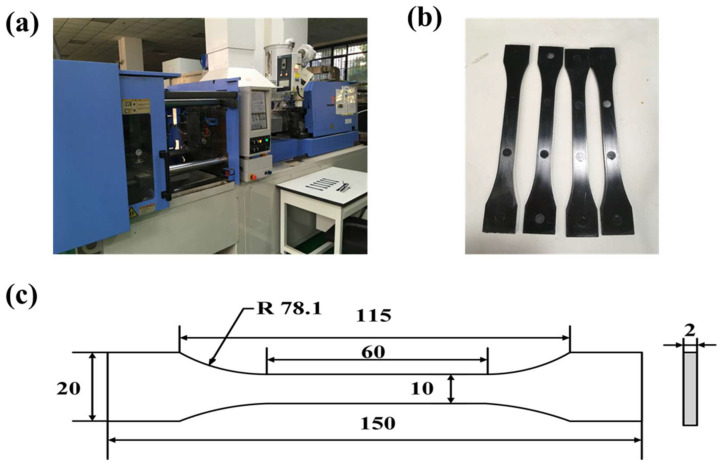
(**a**) All-electric injection molding machine (JSW110AC), (**b**) dumbbell-shaped specimens, and (**c**) dumbbell-shape tensile sample, mm.

Injection molding was carried out using an all-electric injection molding machine (Japan Steel Works, LTD, JSW110AC, Tokyo, Japan), as shown in Figure 1a. The injection molding machine has a screw diameter of 35 mm and a stroke of 140 mm. The maximum injection pressure and holding pressure are 260 MPa and 236 MPa, respectively, and the maximum clamping force is 1370 kN. To generate different thermal histories, an electric mold temperature control unit was employed. The mold temperature was controlled using an STM-910-W water-type mold temperature controller manufactured by Shini Plastics Technologies (Dongguan, China), Inc. The basic working principle of this system is as follows: water in the heating tank is heated and circulated through the mold by a high-pressure centrifugal pump, and then it returns to the heating tank to form a closed-loop cycle. During this process, if the water temperature becomes too high, a temperature sensor sends a signal to the control system, which then activates a solenoid valve to introduce cooling water into the system. When the temperature drops to the set point, the solenoid valve closes, thereby maintaining a constant water temperature throughout the cycle. If the temperature exceeds the upper limit set by the thermal protector (snap-action thermostat), the system triggers an alarm and shuts down to prevent overheating. If the system pressure continues to rise, a safety valve opens to release pressure and ensure operational safety. In addition, if water is lost during operation and the water level drops, a water level switch detects the low level and opens the solenoid valve to automatically refill the tank. Once the appropriate level is reached, the valve closes to prevent the heating tank from running dry. The system employs a PID (Proportional–Integral–Derivative) temperature control algorithm, ensuring stable mold temperature control with a precision of up to ±0.5 °C. In addition, the injection molding machine itself was equipped with multiple real-time sensors (as shown in Supplementary Materials Figure S1). Kistler cavity pressure sensors were employed for in-mold pressure measurement, and these were installed in direct contact with the melt to ensure accurate monitoring during the injection process. The screw position was measured using an MTS Sensors Technology Corporation (Lüdenscheid, Germany) R-series magnetostrictive displacement sensor. The melt pressure inside the barrel, which corresponds to the injection pressure during the molding process, was measured by a Minebea MPC-201-25 pressure sensor installed behind the screw. These sensors allowed for high-precision closed-loop process control, ensuring consistency in cavity filling, packing, and cooling.

The material used in this study was polycarbonate (PC, EXL1414) supplied by SABIC Innovative Plastics (Guangzhou, China). PC has a density of 1.2 × 10^3^ kg/m^3^ and a specific heat capacity of 1750 J/(kg·K). Prior to molding, the material was dried at 393 K for 3 h to remove moisture. The injection molding process parameters are summarized in Table 1, with mold temperatures ranging from 293 K to 333 K. For each mold temperature condition, three tensile specimens were molded, and the mold temperature was monitored between cycles to ensure it returned to the target value before the next shot. It should be noted that 293 K is relatively low for polycarbonate (PC) molding, while 293 K was chosen as the reference mold temperature to represent the lower bound of the mold temperature range, allowing a clear comparison with higher mold temperatures and isolating the effects of thermal history under suboptimal cooling conditions.

Dumbbell-shaped specimens were produced using these parameters, as shown in Figure 1b. It should be noted that the PC was blended with a black masterbatch, which offers good dispersibility and high processing stability. The addition of black masterbatch during PC processing enhances thermal stability, reduces the risk of thermal degradation and deformation, and helps ensure a consistent product quality. The tensile test specimens were prepared in accordance with the GB/T 1040.2-2006 standard [23], had approximate dimensions of 150 mm × 20 mm × 2 mm (Figure 1c), and were stored at room temperature for three days before testing to relieve residual stresses.

### 2.2. Annealing Experiment

To investigate the effect of the thermal history during formation on the deformation behavior of PC, annealing experiments were conducted using specimens molded at a fixed mold temperature of 293 K, as detailed in Table 2. The 293 K condition was selected as a consistent reference to isolate the effect of post-molding annealing. Since this mold temperature introduces minimal thermal relaxation during formation, it provides a clearer baseline for observing annealing effects. During annealing, the specimens were placed in a vacuum oven with temperatures set to 353 K, 373 K, 393 K, and 403 K. At each annealing temperature, samples were extracted at time intervals of 0.2, 0.6, 2, 4, 6, 8, and 23 h to capture the evolution of mechanical properties over time.

### 2.3. Uniaxial Tension Test

**Figure 2:**
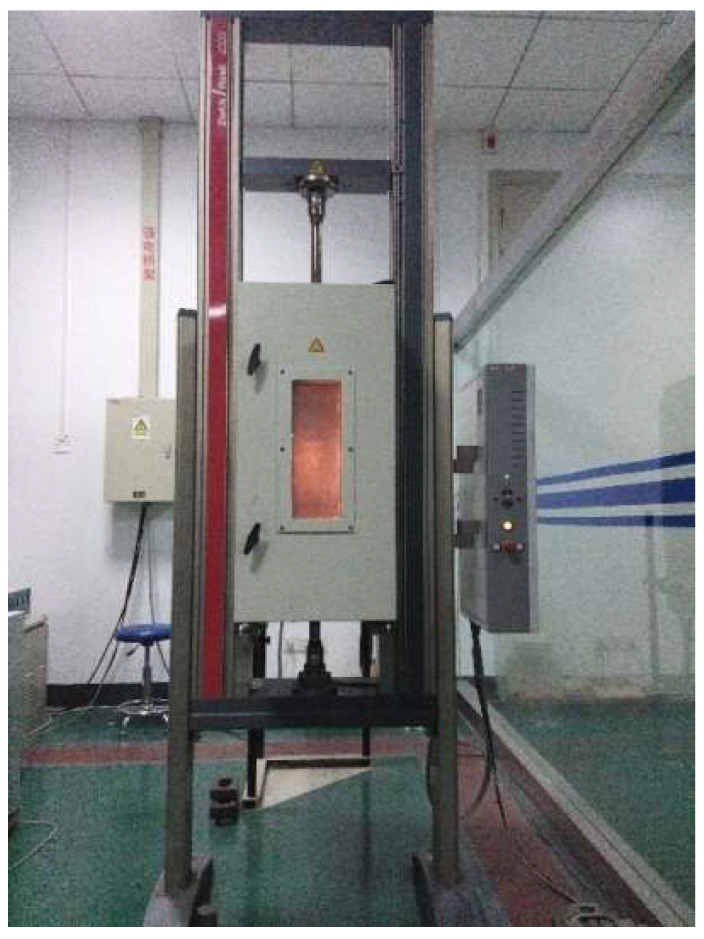
Zwick universal testing machine.

Uniaxial tensile tests were conducted using a Zwick/Roell Z020 universal testing machine equipped with a 10 kN load cell under various conditions, as summarized in Table 3. The entire testing process was controlled and recorded using TestXpert III testing software, ensuring accurate data acquisition and repeatability. The experimental device is equipped with a factory-customized constant-temperature chamber, which is used to study the deformation behavior of polymers at high temperatures, as shown in Figure 2. Annealed PC specimens molded at 293 K were tested at a strain rate of 10^−2^ s^−1^ and a temperature of 293 K. Unannealed specimens molded at the same temperature (293 K) were subjected to tensile tests across different strain rates (10^−3^ s^−1^, 10^−2^ s^−1^, and 10^−1^ s^−1^) and testing temperatures (293 K, 323 K, and 353 K). In addition, unannealed specimens molded at 301 K, 313 K, 323 K, and 333 K were tested at a strain rate of 10^−2^ s^−1^ and temperature of 293 K. Each test condition was repeated at least three times to ensure the reliability and reproducibility of the experimental data.

### 2.4. Constitutive Modeling

**Figure 3:**
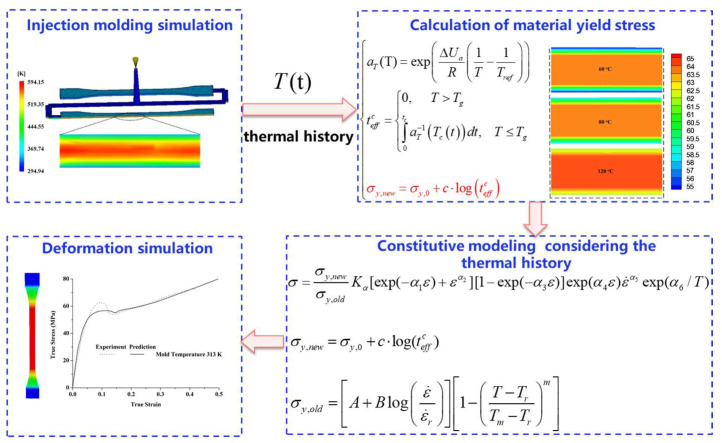
The overall calculation process.

Although prior studies have demonstrated the influence of processing conditions—such as mold temperature, annealing, and cooling rate—on the mechanical behavior of polymers, most efforts have treated the formation process and service behavior as two independent stages. As shown in Figure 3, an integrated framework that directly couples injection molding simulation (via Autodesk Moldflow 2015) with a physically motivated phenomenological constitutive model implemented in ABAQUS/Explicit was proposed. The thermal history during formation, represented by time–temperature data during cooling, is used to compute the equivalent annealing time based on an Arrhenius-type shift factor.

Following the methodology proposed by Govaert and Engels [8,12,13], specimens molded at different temperatures were subjected to annealing for various durations and temperatures, followed by tensile testing. Yield stress versus annealing time curves were plotted for each annealing temperature. These curves were then shifted to form a master curve, which captures the combined effects of annealing temperature and time on the yield stress of the polymer. The shift factor used to construct the master curve was determined using the Arrhenius equation:

(1) $$a(T)=\exp\left(\frac{\Delta U_{a}}{R}(\frac{1}{T}-\frac{1}{T_{ref}})\right)$$

where *ΔU_a_* = 205 kJ/mol [12] represents the activation energy, *R* = 8.314 J/(mol∙K) is the general gas constant, and *T* and *T_ref_* represent the annealing and reference temperature, respectively. The main curve can be fitted by the logarithmic equation, which characterizes the evolution of yield stress during formation and the annealing process:

(2) $$\sigma_{y,new}=\sigma_{y,0}+c\cdot \log\left(t_{eff}+t_{a}\right)$$

where *σ_y_*_,*new*_ is the yield stress, *σ_y_*_,0_ and *c* are the coefficients of the equation, which can be obtained by fitting the main curve, *t_a_* is the equivalent time reflecting the effect of the thermal history during the formation process, and *t_eff_* is related to the thermal history of the annealing process, which represents the integral of the reciprocal of the displacement factor *a*(*T*):

(3) $$t_{eff}=\int_{0}^{t}a^{-1}(T(t))dt$$

Then, the yield stresses in different annealing processes can be obtained according to Equations (3)–(5). During injection molding, the polymer melt is heated to a set temperature in the barrel, enters the flow path through the nozzle, and finally, is cooled and formed in the mold. The polymer melt is cooled to the glass transition temperature (T_g_) and then to the mold temperature. Above T_g_, the modulus of the polymer in the highly elastic state is small (10^0^~10^1^ MPa), while the modulus of the polymer in the glass state (below T_g_) decreases remarkably with decreasing temperature. The effect of temperature on the modulus can be ignored in the highly elastic state, while the effect is significant below T_g_. Therefore, the equivalent time *t_eff_* is the accumulated time from T_g_ to the end of the cooling process, marked as $t_{eff}^{c}$. During the formation thermal history, the displacement factor is

(4) $$a(T)=\exp\left(\frac{\Delta U_{a}}{R}(\frac{1}{T(t)}-\frac{1}{T_{ref}})\right)$$

where *T*(*t*) and *T_ref_* represent the real-time and reference temperatures, respectively. The equivalent time $t_{eff}^{c}$ can be expressed as

(5) $$t_{eff}^{c}=\left\{\begin{array}{c} 0,{\;T>T}_{g}\\ \int_{0}^{t_{e}}a_{T}^{-1}(T(t))dt,\;T\leq T_{g} \end{array}\right\}$$

According to the fitting equation of yield stress, the yield stress during the formation thermal history is obtained:

(6) $$\sigma_{y,new}=\sigma_{y,0}+c\cdot \log\left(t_{eff}^{c}\right)$$

The cooling temperature field during injection molding can be obtained using a commercial software Autodesk Moldflow 2015, then the yield stress for the thermal history during formation *σ_y_*_,*new*_ can be calculated by Equations (7) and (8).

To establish a formation thermal history constitutive model, tensile tests for the specimens prepared at the mold temperature of 293 K were first conducted, then the yield stress was fitted by the equations. A method for fitting the yield stress was proposed based on the Johnson–Cook constitutive model [24]:

(7) $$\sigma_{y,old}=\left[A+B\log(\frac{\dot{\varepsilon }}{{\dot{\varepsilon }}_{r}})\right][1-{\left(\frac{T-T_{r}}{T_{m}-T_{r}}\right)}^{m}]$$

where *A*, *B,* and *m* were the material parameters, ${\dot{\varepsilon }}_{r}$, *T_r_*, and *T_m_* were the reference strain rate, reference temperature, and melting point temperature, respectively, $\dot{\varepsilon }$ and *T* represent the testing strain rate and temperature, and *σ_y_*_,*old*_ is the fitted yield stress.

According to our recent works [20,21,22], phenomenological constitutive models based on the Mulliken–Boyce [25], G’Sell–Jonas [26], and DSGZ [27] models were successfully employed to predict the deformation behavior of PC at low, moderate, and high strain rates for a room temperature of 293 K. This can be decomposed into the α component and the *β* component. The *α* considers the influence of the *α* transition of the material on its mechanical behavior, and the *β* component accounts for the influence of the *β* transition on its mechanical behavior. The detailed descriptions can be found in Ref [20]. To be specific, the constitutive model is expressed as

(8) $$\sigma =w_{\alpha }\sigma_{\alpha }+w_{\beta }\sigma_{\beta }$$

(9) $$\sigma_{a}=K_{a}[\exp(-a_{1}\varepsilon )+\varepsilon^{a_{2}}][1-\exp(-a_{3}\varepsilon )]\exp(a_{4}\varepsilon )\varepsilon^{a_{5}}\exp(a_{6}/T)$$

(10) $$\sigma_{\beta }=K_{\beta }[\exp(-\beta_{1}\varepsilon )+\varepsilon^{\beta_{2}}][1-\exp(-\beta_{3}\varepsilon )]\exp(\beta_{4}\varepsilon )\varepsilon^{\beta_{5}}\exp(\beta_{6}/T)$$

(11) $$w_{a}=E_{a}/(E_{a}+E_{\beta })$$

(12) $$w_{\beta }=E_{\beta }/(E_{a}+E_{\beta })$$

where *E_a_* and *E_β_* are the *α* modulus and the *β* modulus, respectively. *w*_α_ and *w_β_* are the weighting factors of the influences of the *α* and *β* transitions on the mechanical behavior, respectively. Since the deformation behavior of polymers at low strain rates was investigated in this paper, the constitutive model can be simplified to

(13) $$\sigma =K_{a}[\exp(-a_{1}\varepsilon )+\varepsilon^{a_{2}}][1-\exp(-a_{3}\varepsilon )]\exp(a_{4}\varepsilon )\varepsilon^{a_{5}}\exp(a_{6}/T)$$

where *K_a_*, *a*_1_, *a*_2_, *a*_3_, *a*_4_, *a*_5_, and *a*_6_ are the materials coefficients for the constitutive model. To consider the effect of the thermal history during formation on the mechanical behavior of a polymer, a formation thermal history constitutive model was proposed based on the above equations:

(14) $$\;\sigma =\frac{\sigma_{y,new}}{\sigma_{y,old}}K_{a}[\exp(-a_{1}\varepsilon )+\varepsilon^{a_{2}}][1-\exp(-a_{3}\varepsilon )]\exp(a_{4}\varepsilon )\varepsilon^{a_{5}}\exp(a_{6}/T)$$

where *σ_y_*_,*new*_ and *σ_y_*_,*old*_ represent the yield stress for the thermal history during formation and the service process, respectively.

### 2.5. Finite Element Modeling

**Figure 4:**
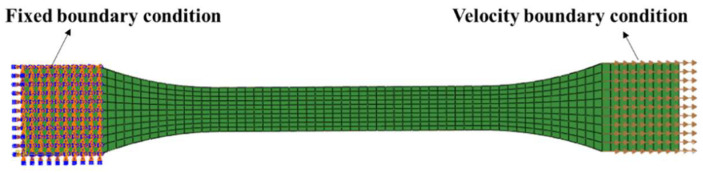
The finite element modeling of a tension specimen.

The constitutive model was implemented in ABAQUS/Explicit through a user material subroutine to predict the deformation behavior of PC, taking the thermal history during formation into account. As shown in Figure 4, the specimen was meshed using eight-node linear reduced integration elements (C3D8R), with a grid division size of 0.5, resulting in a total of 760 elements. The mesh size was recommended by the ABAQUS system to balance computational efficiency and accuracy. A mesh independence study was conducted, and the differences in predicted stress–strain responses were found to be negligible, confirming the suitability of the selected mesh density. As for the boundary conditions, one end of the specimen was fully constrained, while the other end was subjected to a velocity boundary condition to simulate the applied uniaxial tensile deformation. The applied velocity was constant over time. Since the model geometry was relatively small and symmetric simplifications would not yield significant gains, no symmetry constraints were applied to reduce computational effort. To validate the proposed constitutive model, the deformation behavior of specimens at various mold temperatures (293 K, 303 K, 313 K, 323 K) was simulated under different strain rates (10^−1^, 10^−2^, 10^−3^ s^−1^) and temperatures (293 K, 323 K, 353 K).

## 3. Results and Discussion

### 3.1. Experimental Results

**Figure 5:**
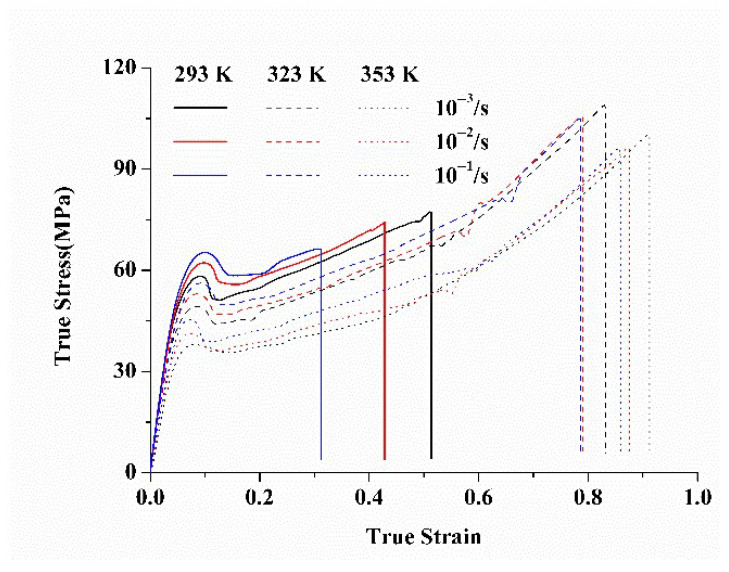
Stress–strain curves of the PC specimens without annealing prepared at the mold temperature of 293 K.

Figure 5 presents the true tensile stress–strain curves of unannealed PC specimens molded at 293 K. The results clearly indicate that the deformation behavior is strongly influenced by both strain rate and temperature. Higher strain rates or lower temperatures lead to an increase in tensile stress. The deformation process of PC can be divided into four distinct stages: elastic deformation, yield, strain softening, and strain hardening. Upon yielding, the molecular chains begin to flow plastically, initiating necking. As deformation continues, the necking region expands along the tensile direction, and the degree of molecular entanglement decreases, leading to pronounced strain softening. With further strain, the molecular chains gradually orient along the stretching direction, resulting in strain hardening. Additionally, the fracture strain decreases as the strain rate increases or temperature decreases, indicating a transition to a stiffer and more brittle material behavior.

**Figure 6:**
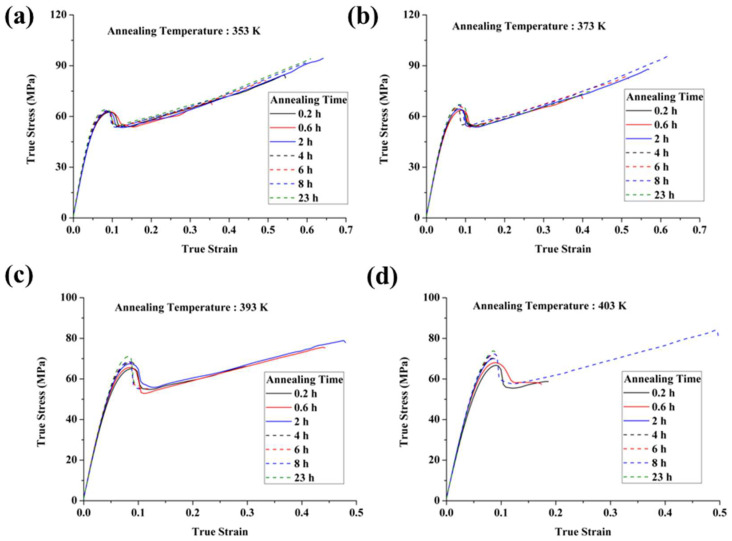
Stress–strain curves of the annealed PC specimens prepared at the mold temperature of 293 K. The annealing temperatures are (**a**) 353 K, (**b**) 373 K, (**c**) 393 K, and (**d**) 403 K, respectively.

Figure 6 presents the true tensile stress–strain curves of annealed PC specimens molded at 293 K, tested at a strain rate of 10^−2^ s^−1^ and a temperature of 293 K. As shown in Figure 6a, annealing at 353 K has a minimal impact on the mechanical properties. At 373 K, the yield stress increases with longer annealing times, while the strain hardening behavior remains largely unaffected. At higher annealing temperatures of 393 K and 403 K, the yield stress increases more significantly with annealing time. However, this increase is accompanied by a reduction in fracture strain, indicating a shift toward a harder and more brittle material.

**Figure 7:**
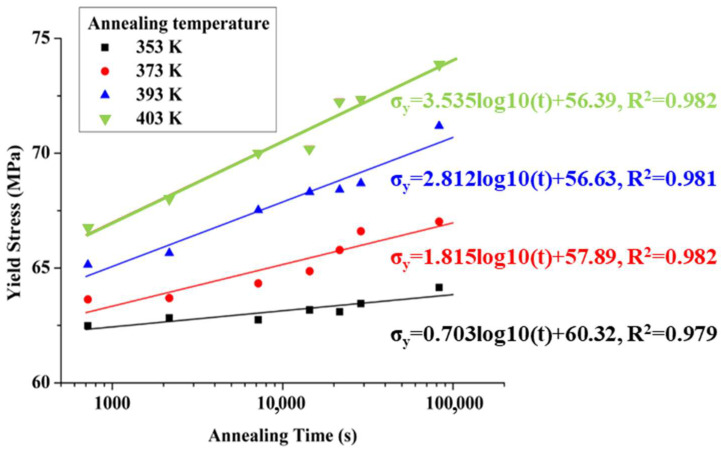
Yield stress of annealed PC specimens prepared at the mold temperature of 293 K.

Table 4 shows the yield stress of PC specimens at different annealing temperatures and times, as derived from Figure 6. The results indicate that yield stress increases with both annealing time and temperature, with a more pronounced increase at higher annealing temperatures. For instance, at an annealing temperature of 353 K, the yield stress increased by approximately 2.67% (from 62.48 to 64.15 MPa), while at 403 K, it increased by about 10.65% (from 66.75 to 73.86 MPa). Figure 7 presents the yield stress of PC specimens at various annealing temperatures and times, with linear fitting applied. The slopes of the curves are 0.703, 1.815, 2.812, and 3.535 at annealing temperatures of 353 K, 373 K, 393 K, and 403 K, respectively. In summary, both higher annealing temperatures and longer annealing times result in increased yield stress, with the yield stress rising more significantly at higher annealing temperatures.

**Figure 8:**
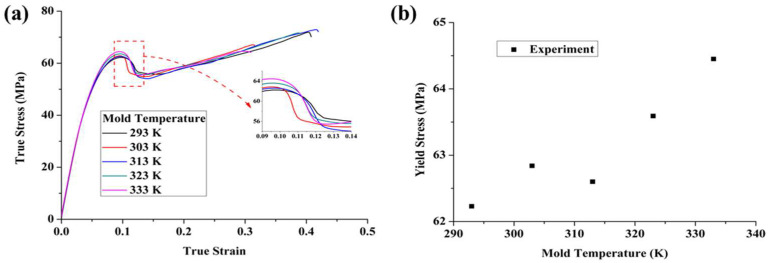
Tensile experimental results of unannealed PC prepared at various mold temperatures: (**a**) stress–strain curves, (**b**) yield stress.

Figure 8 presents the true tensile stress–strain curves of unannealed PC specimens prepared at different mold temperatures, tested at a strain rate of 10^−2^ s^−1^ and a temperature of 293 K. The yield stress increased as the mold temperature rose from 293 K to 333 K, while the deformation process still followed the typical stages: elasticity, yield, strain softening, and strain hardening. As the mold temperature continued to increase, the initial elastic stage remained consistent, indicating that the mold temperature had a negligible effect on the material’s modulus. As shown in Figure 8b, the yield stress increased with higher mold temperatures. Additionally, the strain-hardening stage became more pronounced as the mold temperature increased, suggesting that higher mold temperatures facilitated the relaxation of molecular chains, making it easier for the chains to orient, which in turn led to greater strain hardening. It is worth noting that the yield stress at 303 K is slightly higher than that at 313 K. This may be attributed to microstructural variations or local differences in thermal history and residual stress at lower mold temperatures, which can have a nonlinear influence on yield behavior in amorphous thermoplastics.

### 3.2. Model Predictions

#### 3.2.1. Solution for Model Parameters

**Figure 9:**
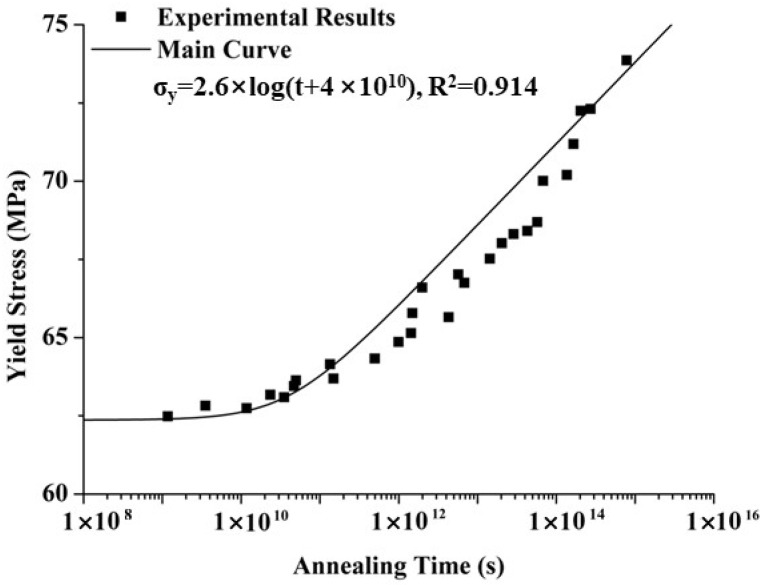
Experimental and predicted results of yield stress of PC in the main curve.

The yield stress of annealed PC specimens prepared at a mold temperature of 293 K was initially processed using the yield stress calculation method for the formation process. As shown in Figure 9, the main curves for the yield stress of PC were plotted using Equation (3), with ∆Ua = 205 kJ/mol, R = 8.314 J/(mol·K), and Teff = 293 K. When the annealing time was less than 1010 s, the yield stress changed only slightly; however, it increased significantly once the annealing time exceeded 1010 s. It should be noted that the equivalent time value (1010 s) reflects the logarithmic fitting used in the master curve construction based on time–temperature superposition. Though such long times are not physical, they are mathematically required for curve fitting across the entire temperature range. A logarithmic equation $\sigma_{y,new}=\sigma_{y,0}+c\cdot \log\left(t_{eff}+t_{a}\right)$ was then used to fit the main curve, with the values *σ*_*y*,0_ = 34.8 MPa, *c* = 2.6 MPa, and *t_a_* = 4 × 10^10^ s obtained via the particle swarm method. The fitted main curve closely matched the experimental main curve, indicating that the yield stress evolution, derived through this method, accurately reflects the yield stress of PC specimens undergoing the actual annealing process.

After fitting the main curve of yield stress, Autodesk Moldflow 2015 was used to simulate the cooling history of injection molding at different mold temperatures. The ambient temperature was set to 293 K, and the melt temperature was set to 593 K. The cooling time was 60 s, with mold temperatures set at 293 K, 303 K, 313 K, 323 K, and 333 K.

**Figure 10:**
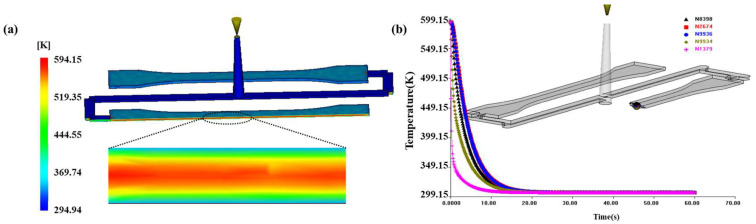
Cooling results of the tensile specimen of PC injected at a mold temperature of 303 K: (**a**) cooling field chart, (**b**) cooling history.

The mold temperature is not constant during the injection molding cycle due to heat exchange with the molten polymer. The coolant gallery temperature was maintained at 293 K, 303 K, 313 K, 323 K, and 333 K. In the Autodesk Moldflow simulations, the effects of the electric heating rods used in the experiments were not explicitly modeled. Instead, the simulation relied on specifying the initial melt temperature, ambient conditions, coolant (cooling channel) temperatures, and mold material properties to approximate the overall thermal behavior during the injection molding process. Figure 10 shows the distribution of temperature and cooling history at a mold temperature of 303 K. As shown in Figure 10b, the surface layer of the specimen, in contact with the mold, cooled rapidly (N1379), while the core layer cooled more slowly (N9936). Additionally, the cooling rate of the intermediate nodes (N8393, N2674, and N9934) was between that of the surface and core nodes.

**Figure 11:**
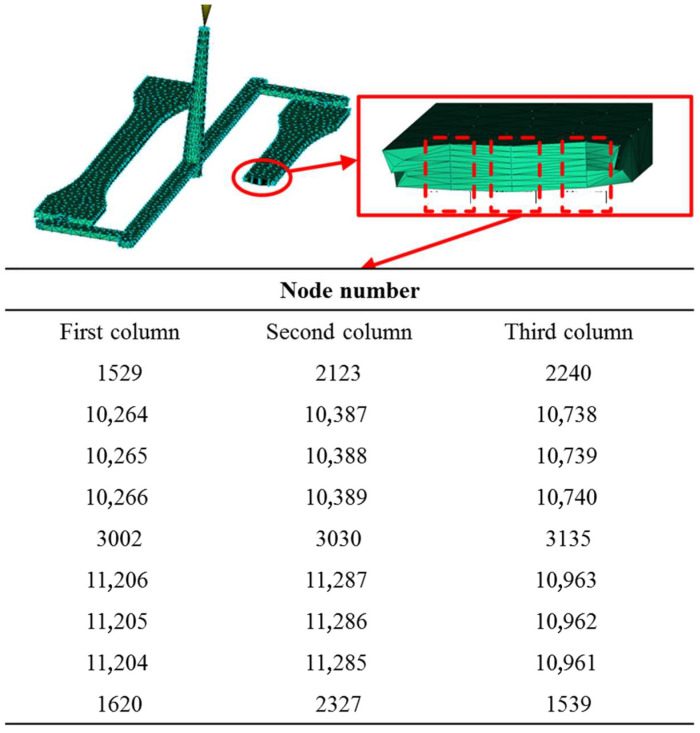
Selected nodes with increasing thickness.

**Figure 12:**
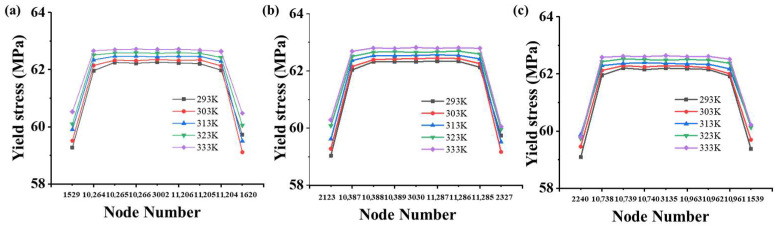
Yield stresses of nodes in the (**a**) first column, (**b**) second column, and (**c**) third column while processing at various mold temperatures.

Since the cooling rate of the surface layer was faster than that of the core layer, this resulted in a lower effective temperature $t_{eff}^{c}$ for the surface layer. Consequently, the yield stress of the surface nodes was lower than that of the core nodes. As shown in Figure 11, nodes with increasing thickness were selected, and their yield stress was calculated using Equations (6)–(8). As shown in Figure 12, the yield stress of the core layer was higher than that of the surface layer, and the yield stress increased with higher mold temperatures. The detailed results are presented in Tables S1–S3 (see Supporting Information, Section S1). It is evident that the yield stress of the core layer was higher than that of the surface layer, and the yield stress increased with higher mold temperatures.

**Figure 13:**
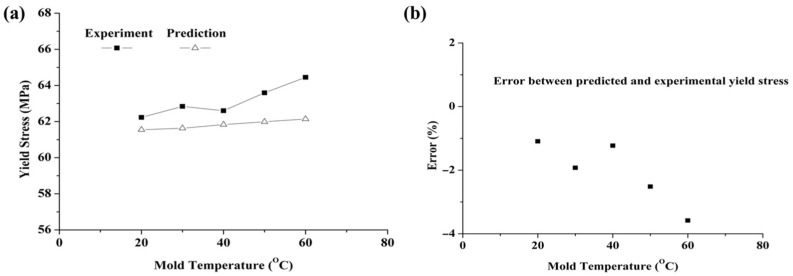
Predicted and experimental yield stresses at various mold temperatures: (**a**) comparison between predicted and experimental values, (**b**) error.

According to the methods proposed by Govaert and Engels [8,12,13], the simulated yield stress was calculated as the average yield stress of the nodes with increasing thickness. The predicted yield stresses at mold temperatures of 293 K, 303 K, 313 K, 323 K, and 333 K were 61.55 MPa, 61.63 MPa, 61.83 MPa, 61.99 MPa, and 62.14 MPa, respectively. As shown in Figure 13a, the predicted yield stresses closely matched the experimental values. Furthermore, the error was calculated as the relative deviation between the experimental yield stress and the model-predicted values. As shown in Figure 13b, the error was less than 5%, demonstrating that the proposed method can predict how the thermal history of the injection-molded component influences the yield stress. Meanwhile, the underestimation of yield stress, particularly at higher mold temperatures, can be attributed to certain limitations of the proposed model. These include the simplified representation of heat transfer within the mold, especially the omission of explicit modeling of the electric heating rods and cooling channels, which may result in discrepancies between the simulated and actual thermal history.

Then, the material coefficients for the phenomenological constitutive model (K*_a_*, *a*_1_, *a*_2_, *a*_3_, *a*_4_, *a*_5_, and *a*_6_) were determined by the stress–strain in Figure 3 with the method described in our recent work [20] (see detailed description in Supporting Information, Section S2). Combined with above parameters related to the thermal history during formation, the material coefficients of PC are shown in Table 5.

#### 3.2.2. Prediction Results

**Figure 14:**
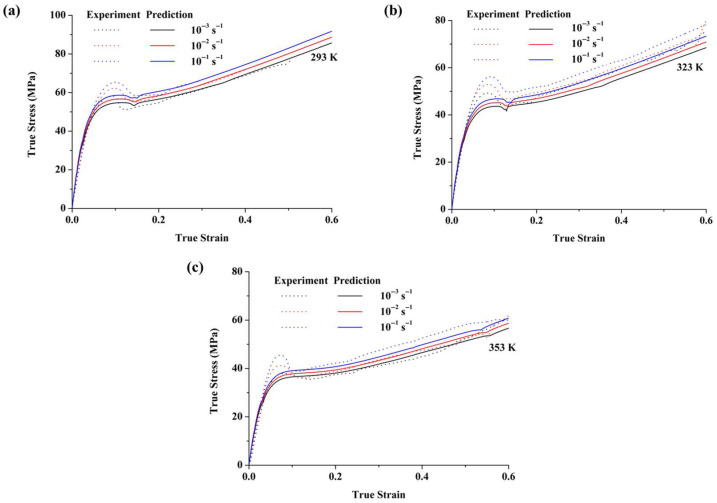
Predicted and experimental results of the large deformation of PC specimens at various strain rates and temperatures when injected at room temperature: (**a**) 293K, (**b**) 323K, and (**c**) 353K.

Figure 14 shows the predicted and experimental results for the large deformation behavior of PC specimens at various strain rates and temperatures, prepared at room temperature. The results indicate that the new constitutive model accurately describes the initial elastic stage, subsequent yielding, and strain-softening and strain-hardening behaviors. It also effectively captures the rate-dependent and temperature-dependent deformation characteristics. Additionally, the model successfully predicts large deformation behavior at different strain rates, with the predicted strain hardening aligning well with experimental data. However, the accuracy of the predicted yield stress is somewhat limited. This is primarily due to the fact that the phenomenological constitutive model cannot fully capture the steep strain-softening behavior, leading to a slight underestimation of the yield stress, particularly at higher mold temperatures. These include the simplified treatment of heat transfer and the assumption of a uniform thermal history within individual layers, which may not fully capture the actual thermal gradients during the molding process.

**Figure 15:**
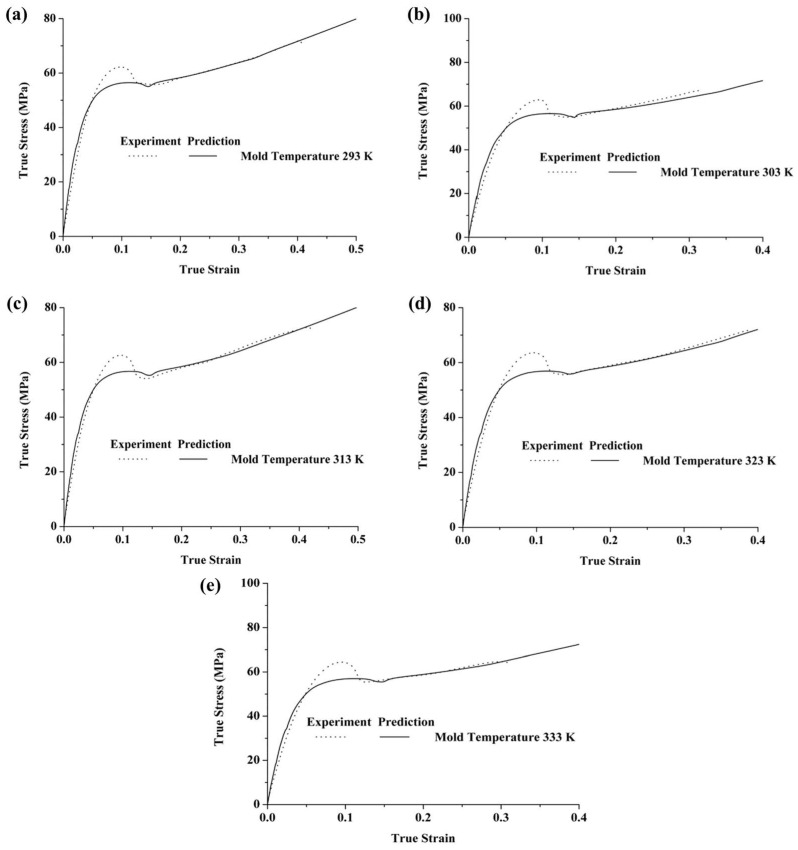
Predicted vs. experimental true stress–strain curves of PC specimens tested at 293 K and a strain rate of 10^−2^ s^−1^, injected at mold temperatures of (**a**) 293 K, (**b**) 303 K, (**c**) 313 K, (**d**) 323 K, and (**e**) 333 K.

Figure 15 shows the predicted and experimental results of the deformation behaviors of PC specimens at different mold temperatures under service conditions (strain rate = 10^−2^ s^−1^, temperature = 293 K). The results demonstrate that the constitutive model, which considers the thermal history during formation, effectively describes the initial elastic stage, subsequent yielding, and strain-softening and final strain-hardening behaviors of PC specimens. Regarding the influence of mold temperature, it does not fundamentally change the stress–strain trend of polycarbonate. As the mold temperature increases, the yield stress exhibits only minor variations. The proposed model is formulated to capture the typical deformation behavior of injection-molded PC under different mold temperatures, including the initial nonlinear elasticity, subsequent yield, strain softening, and eventual strain hardening. The model predictions consistently reproduce these mechanical responses across all tested conditions, although the variation in yield stress with mold temperature is limited.

## 4. Conclusions

The mechanical behavior of PC was experimentally investigated, and a constitutive model considering the thermal history during formation was proposed. The following conclusions can be drawn:

(1) During the injection molding process, a slower cooling rate or higher mold temperature results in higher yield stress, with the core layer exhibiting greater yield stress than the surface layer. During the annealing process, longer annealing times and higher annealing temperatures contribute to higher yield stress. In the service process, higher strain rates and lower temperatures lead to increased yield stress.
(2) A formation thermal history-based constitutive model was developed to study the mechanical behavior of PC. Simulation of the formation process showed that the predicted yield stress of PC specimens at various mold temperatures closely matched experimental values. The absolute value of the error between the simulated value and the experimental value is less than 5%, indicating that the model can accurately simulate the yield stress in the thermal history during formation. In the service process, the new phenomenological model accurately predicted large deformation behavior at different strain rates and temperatures. The formation thermal history constitutive model can precisely capture the large deformation behavior of PC specimens prepared at various mold temperatures, including initial elasticity, strain hardening, yield deformation, and strain softening.

In summary, the proposed formation thermal history-based constitutive model accurately predicts the large deformation behavior of PC tensile specimens under various molding conditions. It enables an integrated simulation framework that connects injection molding with in-service mechanical performance, addressing the long-standing gap between formation process simulation and service behavior prediction. However, the current study focuses on relatively simple geometries. Future work will extend this integrated modeling approach to more complex structural components to further validate its applicability and robustness.

## Figures and Tables

**Table 1:** Injection molding process parameters.

Injection Temperature (K)	Volume Flow Rate (cm^3^/s)	Injection Pressure (MPa)	Hold Pressure (MPa)	Hold Time (s)	Cooling Time (s)	Mold Temperature (K)
593	36.75	147	117.6	8	60	293, 303, 313, 323, 333

**Table 2:** Annealing experiments.

Mold Temperature (K)	Annealing Temperature (K)	Annealing Time (h)
293	353	0.2, 0.6, 2, 4, 6, 8, 23
	373	0.2, 0.6, 2, 4, 6, 8, 23
	393	0.2, 0.6, 2, 4, 6, 8, 23
	403	0.2, 0.6, 2, 4, 6, 8, 23

**Table 3:** The uniaxial tensile tests.

Mold Temperature (K)	Annealing	Strain Rate (s^−1^)	Temperature (K)
293	Yes	10^−2^	293
293	No	10^−3^, 10^−2^, 10^−1^	293, 323, 353
303, 313, 323, 333	No	10^−2^	293

**Table 4:** Yield stress of PC at various annealing temperatures and times.

Time (h)	Yield Stress at 353 K (MPa)	Yield Stress at 393 K (MPa)	Yield Stress at 393 K (MPa)	Yield Stress at 403 K (MPa)
0.2	62.48	63.63	65.14	66.75
0.6	62.82	63.69	65.65	68.02
2	62.74	64.33	67.52	70.01
4	63.17	64.86	68.31	70.20
6	63.09	65.78	68.41	72.25
8	63.45	66.60	68.69	72.31
23	63.45	67.02	71.19	73.86

**Table 5:** Material coefficients of PC.

Material Coefficient		Value
Constitutive model	$K_{\alpha }$	$10.55\;\mathrm{MPa}\cdot {\mathrm{ms}}^{\alpha_{5}}$
	$\alpha_{1}$	11.91
	$\alpha_{2}$	0.459
	$\alpha_{3}$	19.29
	$\alpha_{4}$	0.209
	$\alpha_{5}$	0.0149
	$\alpha_{6}$	705.8 K
Yield stress	*A*	58.23 MPa
	*B*	3.54 MPa
	*m*	0.777
Processing history	$\Delta U_{a}$	205,000 J/mol
	*R*	8.314 J/(mol·K)
	*T_r_*	293 K
	*T_m_*	553 K
	$\sigma_{y,0}$	34.8 MPa
	*c*	2.6 MPa

## Data Availability

Data are contained within the article and Supplementary Materials.

## Supplementary Materials

The following supporting information can be downloaded at https://www.mdpi.com/article/10.3390/polym17152096/s1, Figure S1: Schematic of sensor integration in the injection molding system, including mold cavity sensors, pressure sensor, screw position sensor, and mold temperature controller; Table S1: Yield stress of nodes in the first column processing at various mold temperatures; Table S2: Yield stress of nodes in the second column processing at various mold temperatures; Table S3: Yield stress of nodes in the third column processing at various mold temperatures.
