# Supplementary material for: Thermal History-Dependent Deformation of Polycarbonate: Experimental and Modeling Insights
Source: Polymers (Basel). 2025 Jul 30;17(15):2096. doi: 10.3390/polym17152096 (PMC12349233; doi:10.3390/polym17152096)
Supplement: Supplementary file 1 [file polymers-17-02096-s001.zip › polymers-3734619-supplementary.pdf]

Supplementary Information

Thermal History–Dependent Deformation of Polycarbonate: Experimental and Modeling Insights

Maoyuan Li [a, b], Haitao Wang[b], Guancheng Shen*[b,c], Tianlun Huang*[b,d],Yun Zhang *[b]

[a] School of Aerospace Engineering, Xiamen University, Xiamen, 361005, China

[b] State key Laboratory of Materials Processing and Die & Mold Technology, School of Materials Science and Engineering, Huazhong University of Science and Technology, Wuhan, 430074, China

[c] Xi' an Modern Chemistry Research Institute, Xi' an 710065, China;

[d]Shenzhen Institutes of Advanced Technology, Chinese Academy of Sciences, Shenzhen, 518055, Guangdong, China

* Corresponding author: marblezy@hust.edu.cn (Yun Zhang); tl.huang1@siat.ac.cn (Tianlun Huang); gc_shen@outlook.com (Guancheng Shen)

S1. Sensor integration in the injection molding system

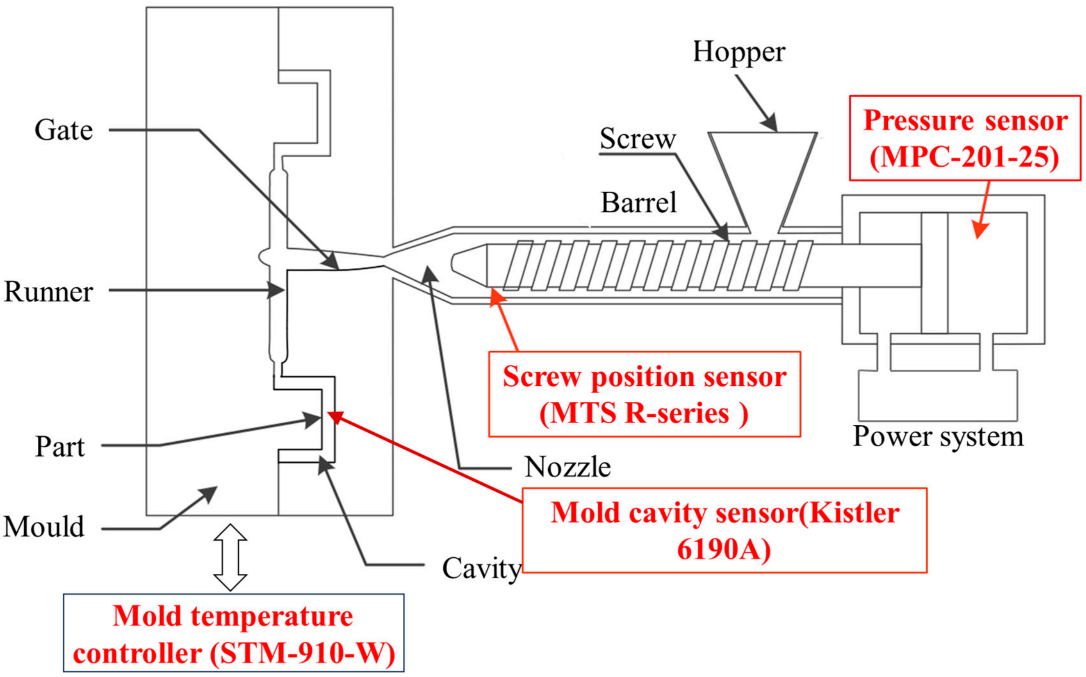

Figure S1 Schematic of sensor integration in the injection molding system, including mold cavity sensors, pressure sensor, screw position sensor, and mold temperature controller

S2. The Yield stress of nodes in different column

Table S1 Yield stress of nodes in the first column processing at various mold temperatures.

Node number	Yield stress (MPa)				
	293 K	303 K	313 K	323 K	333 K
1529	59.2755	59.5105	59.904	60.0933	60.5253
10264	61.9643	62.1471	62.3408	62.5134	62.6583
10265	62.253	62.3299	62.4703	62.5813	62.7019

10266	62.2238	62.3165	62.4691	62.5902	62.719
3002	62.2617	62.3529	62.4481	62.5732	62.7064
11206	62.2367	62.3243	62.4729	62.5903	62.7162
11205	62.2096	62.3412	62.4706	62.5723	62.6858
11204	61.9828	62.131	62.2888	62.4273	62.637
1620	59.714	59.1099	59.5	60.0531	60.4714

Table S2 Yield stress of nodes in the second column processing at various mold temperatures.

Node	Yield stress (MPa)				
number	293 K	303 K	313 K	323 K	333 K
2123	59.0353	59.2754	59.6121	60.0783	60.2875
10387	62.0337	62.155	62.3673	62.5095	62.6885
10388	62.3216	62.3921	62.5337	62.659	62.8035
10389	62.3224	62.418	62.5268	62.6715	62.7902
3030	62.3264	62.4299	62.5454	62.6494	62.8197
11287	62.3412	62.4454	62.5612	62.6647	62.7925
11286	62.3344	62.434	62.5463	62.6928	62.8117
11285	62.1285	62.2568	62.4222	62.5907	62.7906
2327	59.7371	59.1671	59.5109	59.9581	60.0555

Table S3 Yield stress of nodes in the third column processing at various mold temperatures.

Node number	Yield stress (MPa)				
	293 K	303 K	313 K	323 K	333 K
2240	59.084	59.4575	59.8767	59.7363	59.7928
10738	61.9541	62.1222	62.2918	62.4319	62.5836
10739	62.2068	62.2889	62.3676	62.524	62.6216
10740	62.1518	62.2458	62.3946	62.5007	62.607
3135	62.198	62.2871	62.372	62.4801	62.6368
10963	62.1854	62.2683	62.3479	62.5062	62.6061
10962	62.1575	62.2186	62.3422	62.4832	62.6178
10961	61.9232	62.0072	62.178	62.3741	62.5151
1539	59.3699	59.6944	60.2115	60.1238	60.223

S3. Solution for material coefficients

Since the deformation behavior of polymers at low strain rates was investigated in this paper, the constitutive model can be simplified to:

$$\sigma_a = K_a[\exp(-a_1\varepsilon)+\varepsilon^{a_2}][1-\exp(-a_3\varepsilon)]\exp(a_4\varepsilon)\varepsilon^{a_5}\exp(a_6/T) \quad \text{(S1)}$$

By selecting points of the same large strain, (ε, σ_1) and (ε, σ_2), on two true stress–strain curves with different low strain rates ($\dot{\varepsilon}_1$, $\dot{\varepsilon}_2$) but the same temperature, α_5 is calculated:

$$\alpha_5 = \frac{\ln(\sigma_1/\sigma_2)}{\ln(\dot{\varepsilon}_1/\dot{\varepsilon}_2)} \quad \text{(S2)}$$

By selecting points of the same large strain, (ε, σ_1) and (ε, σ_2), on two curves with

different temperatures (T_1, T_2) but the same low strain rate, α_6 is obtained:

$$\alpha_6 = \frac{\ln(\sigma_1 / \sigma_2)}{1/T_1 - 1/T_2} \tag{S3}$$

By selecting most of the points on one curve with a low strain rate and using particle swarm optimization (PSO), K_α, α_1, α_2, α_3, and α_4 can be determined.

S4. VUMAT for proposed model

```
      subroutine vumat(
C Read only (unmodifiable)variables -
     1  nblock, ndir, nshr, nstatev, nfieldv, nprops, lanneal,
     2  stepTime, totalTime, dt, cmname, coordMp, charLength,
     3  props, density, strainInc, relSpinInc,
     4  tempOld, stretchOld, defgradOld, fieldOld,
     5  stressOld, stateOld, enerInternOld, enerInelasOld,
     6  tempNew, stretchNew, defgradNew, fieldNew,
C Write only (modifiable) variables -
     7  stressNew, stateNew, enerInternNew, enerInelasNew )
C
      include 'vaba_param.inc'
C

      dimension props(nprops), density(nblock), coordMp(nblock,*),
     1  charLength(nblock), strainInc(nblock,ndir+nshr),
     2  relSpinInc(nblock,nshr), tempOld(nblock),
     3  stretchOld(nblock,ndir+nshr),
     4  defgradOld(nblock,ndir+nshr+nshr),
     5  fieldOld(nblock,nfieldv), stressOld(nblock,ndir+nshr),
     6  stateOld(nblock,nstatev), enerInternOld(nblock),
     7  enerInelasOld(nblock), tempNew(nblock),
     8  stretchNew(nblock,ndir+nshr),
     8  defgradNew(nblock,ndir+nshr+nshr),
     9  fieldNew(nblock,nfieldv),
     1  stressNew(nblock,ndir+nshr), stateNew(nblock,nstatev),
     2  enerInternNew(nblock), enerInelasNew(nblock)
C
      character*80 cmname
C
```

```
      double precision:: KAlpha,Alpha1,Alpha2,Alpha3,Alpha4,Alpha5,
     1                        Alpha6
      double precision:: KBeta,Beta1,Beta2,Beta3,Beta4,Beta5,Beta6
      double precision:: toler,con
      double precision:: emod,enu,temp,eg,eg2,eg3,eg6,ebulk3,elam
      double precision:: trace,smean,sig1,sig2,sig3,sig4,sig5,sig6,
     1                         sig11,sig22,sig33
      integer::newton
      double precision::smisesold,eqplas,strmean,str1,str2,str3,str4,
     1            str5,str6,equstrainNew,equstrainold,deqpl,syiel0,shydr0
      double precision::syield,rhs,hard,smises
      double precision,dimension(6):: eelas,eplas,flow
      parameter (one=1.0D0,two=2.0D0,three=3.0D0,six=6.0D0, half =0.5d0,
     1      third=1.d0/3.d0)
```

! the following codes is for debuging,only input a number one time in the dos window

```
!        logical, save :: FirstCall=.true.
!        integer :: dummyVar
!        if (FirstCall==.true.) then
!           FirstCall = .false.
!           read(*,*) dummyVar
!        end if

      newton=40
      toler=1.0d-6
      con=sqrt(2.d0/3.d0)
C
C state(*,1)=elastic strain component 11
C state(*,2)=elastic strain component 22
C state(*,3)=elastic strain component 33
C state(*,4)=elastic strain component 12
C state(*,5)=elastic strain component 23
C state(*,6)=elastic strain component 31
C
C state(*,7)=plastic strain component 11
C state(*,8)=plastic strain component 22
C state(*,9)=plastic strain component 33
C state(*,10)=plastic strain component 12
C state(*,11)=plastic strain component 23
C state(*,12)=plastic strain component 31
C
```

```
C state(*,13)=equivalent plastic strain
C
C state(*,14)=total strain component 11
C state(*,15)=total strain component 22
C state(*,16)=total strain component 33
C state(*,17)=total strain component 12
C state(*,18)=total strain component 23
C state(*,19)=total strain component 31
C
C state(*,20)=equivalent strain

C
C state(*,21)=total stress component 11
C state(*,22)=total stress component 22
C state(*,23)=total stress component 33
C state(*,24)=total stress component 12
C state(*,25)=total stress component 23
C state(*,26)=total stress component 31
C
C state(*,27)==smises stress


C       seven parameters of the New model

        KAlpha=props(1)
        Alpha1=props(2)
        Alpha2=props(3)
        Alpha3=props(4)
        Alpha4=props(5)
        Alpha5=props(6)
        Alpha6=props(7)

        KBeta=props(8)
        Beta1=props(9)
        Beta2=props(10)
        Beta3=props(11)
        Beta4=props(12)
        Beta5=props(13)
        Beta6=props(14)

C       props(1):KAlpha
C       props(2):Alpha1
C       props(3):Alpha2
```

```
C       props(4):Alpha3
C       props(5):Alpha4
C       props(6):Alpha5
C       props(7):Alpha6
C       props(8):KBeta
C       props(9):Beta1
C       props(10):Beta2
C       props(11):Beta3
C       props(12):Beta4
C       props(13):Beta5
C       props(14):Beta6
C
C       props(15) - Alpha component of Young's Modulus
C       props(16) - Beta component of Young's Modulus
C       props(17) - possion radio
C       props(18) - temperature
C       props(19) - strain rate
C       props(20) - new yield stress

        emod=props(15)+props(16)
        enu=props(17)
        temp=props(18)

        eg=emod/(one+enu)/two
        eg2=eg*two
        eg3=eg*three
        eg6=eg*six
        ebulk3=emod/(one-two*enu)
        elam=(ebulk3-eg2)/three
!     the same
!       elam=eg2*(emod-eg2)/(eg6-two*emod)

        if ( stepTime .eq. zero ) then
            do i = 1, nblock
            trace = strainInc(i,1) + strainInc(i,2) + strainInc(i,3)
            stressNew(i,1) = stressOld(i,1)
     *          + eg2 * strainInc(i,1) + elam * trace
            stressNew(i,2) = stressOld(i,2)
     *          + eg2 * strainInc(i,2) + elam * trace
            stressNew(i,3) = stressOld(i,3)
     *          + eg2 * strainInc(i,3) + elam * trace
            stressNew(i,4)=stressOld(i,4) + eg2 * strainInc(i,4)
            stressNew(i,5)=stressOld(i,5) + eg2 * strainInc(i,5)
```

```
          stressNew(i,6)=stressOld(i,6) + eg2 * strainInc(i,6)

          end do
      else


      do   i=1,nblock
         trace=strainInc(i,1)+strainInc(i,2)+strainInc(i,3)

C define stress component
         stressNew(i,1) = stressOld(i,1)
     *        + eg2 * strainInc(i,1) + elam * trace
         stressNew(i,2) = stressOld(i,2)
     *        + eg2 * strainInc(i,2) + elam * trace
         stressNew(i,3) = stressOld(i,3)
     *        + eg2 * strainInc(i,3) + elam * trace
         stressNew(i,4)=stressOld(i,4) + eg2 * strainInc(i,4)
         stressNew(i,5)=stressOld(i,5) + eg2 * strainInc(i,5)
         stressNew(i,6)=stressOld(i,6) + eg2 * strainInc(i,6)

C        calculate the current smises stress
         sig1=stressOld(i,1)+elam*trace+eg2*strainInc(i,1)
         sig2=stressOld(i,2)+elam*trace+eg2*strainInc(i,2)
         sig3=stressOld(i,3)+elam*trace+eg2*strainInc(i,3)
         sig4=stressOld(i,4) +eg2*strainInc(i,4)
         sig5=stressOld(i,5) +eg2*strainInc(i,5)
         sig6=stressOld(i,6) +eg2*strainInc(i,6)

         smean=(sig1+sig2+sig3)/3
         sig11=sig1-smean
         sig22=sig2-smean
         sig33=sig3-smean



smisesold=(one/con)*sqrt(sig11**2+sig22**2+sig33**2+two*sig4**2
     1       +two*sig5**2+two*sig6**2)

         stateNew(i,28)=smisesold
         do k1=1,6
           eelas(k1)=stateOld(i,k1)+strainInc(i,k1)
           eplas(k1)=stateOld(i,k1+6)
           stateNew(i,k1+13)=stateOld(i,k1+13)+strainInc(i,k1)
```

```
      enddo

      eqplas=stateOld(i,13)

C      calculate equivalent strain
      strmean=(stateNew(i,14)+stateNew(i,15)+stateNew(i,16))/3
      str1=stateNew(i,14)-strmean
      str2=stateNew(i,15)-strmean
      str3=stateNew(i,16)-strmean
      str4=stateNew(i,17)
      str5=stateNew(i,18)
      str6=stateNew(i,19)


c      equstrain:equivalent strain
      equstrainNew=con*sqrt(str1**2+str2**2+str3**2+two*str4**2+
     1       two*str5**2+two*str6**2)

      equstrainold=stateOld(i,20)
      stateNew(i,20)=equstrainNew


C      call userhard subroutine, get harding rate and yield stress
      !deqpl=1.d-13
      call userhard(syiel0,hard,props(1),equstrainNew,
     1                 equstrainold,dt)
      stateNew(i,33)=syiel0
      if((syiel0>1.0d-8) .and. (stateOld(i,29)==0)) then
        stateOld(i,29)=1
      endif
      stateNew(i,29)=stateOld(i,29)
C
C      determine whether yielding is actived
C
      if((stateNew(i,29)==1) .and. (smisesold>(1+toler)*syiel0)) then
C
C        if plastic deformation has occured,   determine the flow direction
C
        shydr0=(stressNew(i,1)+stressNew(i,2)+stressNew(i,3))/3.d0

        do k1=1,3
          flow(k1)=(stressNew(i,k1)-shydr0) /smisesold
        enddo
```

```
            do k1=4,6
                flow(k1)=stressNew(i,k1) / smisesold
            enddo

C           begin newton iteration
            syield = syiel0
            deqpl=1.d-13
            do knewton=1,newton

                rhs=smisesold-eg3*deqpl-syield
                deqpl=deqpl+rhs/(eg3+hard)

                call userhard(syield,hard,props(1),equstrainNew+deqpl,
     1                            equstrainold,dt)

                if(abs(rhs) < toler*syiel0) goto 140

            enddo
 140        continue

C           update stress and strain
C           whether the following codes is true?divited by 2?
            do k1=1,3
                stressNew(i,k1) = flow(k1)*syield+shydr0
                eelas(k1) = eelas(k1)-three * flow(k1)*deqpl/two
                eplas(k1) = eplas(k1)+three * flow(k1)*deqpl/two
            enddo

            do k1=4,6
                stressNew(i,k1) = flow(k1)*syield
                eelas(k1) = eelas(k1)-three * flow(k1)*deqpl
                eplas(k1) = eplas(k1)+three * flow(k1)*deqpl
            enddo

            eqplas=eqplas+deqpl

          endif

          smises=(stressNew(i,1)-stressNew(i,2)) *
     1                (stressNew(i,1)-stressNew(i,2)) +
     2            (stressNew(i,2)-stressNew(i,3)) *
     3                (stressNew(i,2)-stressNew(i,3)) +
     4            (stressNew(i,3)-stressNew(i,1)) *
```

```fortran
     5                    (stressNew(i,3)-stressNew(i,1))
        do   k1=4,6
             smises=smises+six*stressNew(i,k1)*stressNew(i,k1)
        enddo
        smises=sqrt(smises/two)

        do   k1=1,6
             stateNew(i,k1) = eelas(k1)
             stateNew(i,k1+6) = eplas(k1)
        enddo

        do   k1=1,6
             stateNew(i,20+k1) = stressNew(i,k1)
        enddo
        stateNew(i,13)=eqplas
        stateNew(i,27)=smises
        stateNew(i,40) = abs(equstrainNew+deqpl-equstrainold)/dt

        stressPower = half * (
     *     ( stressOld(i,1) + stressNew(i,1) ) * strainInc(i,1) +
     *     ( stressOld(i,2) + stressNew(i,2) ) * strainInc(i,2) +
     *     ( stressOld(i,3) + stressNew(i,3) ) * strainInc(i,3) ) +
     *     ( stressOld(i,4) + stressNew(i,4) ) * strainInc(i,4) +
     *     ( stressOld(i,5) + stressNew(i,5) ) * strainInc(i,5) +
     *     ( stressOld(i,6) + stressNew(i,6) ) * strainInc(i,6)

        enerInternNew(i) = enerInternOld(i) + stressPower / density(i)
C
C Update the dissipated inelastic specific energy -
C
        plasticWorkInc = half * ( syiel0+syield ) * deqpl
        enerInelasNew(i) = enerInelasOld(i)
     *     + plasticWorkInc / density(i)
      enddo

      endif
      return
      end

C
C
C
```

```
      subroutine userhard(syield,hard,table,strainNew,strainOld,dt)
C
C      syield: yield stress
C      hard: hard coefficient
C      table: element inputting parameters
C      strainNew:    strain at the t+dt time
C      strainOld:    strain    at the t time
C      dt: time increments
C
C
      include 'vaba_param.inc'
      double precision,dimension(20):: table
      double precision:: KAlpha, Alpha1, Alpha2, Alpha3, Alpha4, Alpha5,
     1                                  Alpha6
      double precision:: KBeta, Beta1, Beta2, Beta3, Beta4, Beta5, Beta6
      double precision:: AlphaModulus, BetaModulus, temp
      double precision:: HAlpha1, HAlpha2, HBeta1, HBeta2, hard
      double precision:: syieldAlpha, syieldBeta, syield
      double precision:: NorminalStrainrate, NewYieldStress,
     1                                OldYieldStress
      double precision::A, B, Eref,Tm,Tr,m
C
      KAlpha=table(1)
      Alpha1=table(2)
      Alpha2=table(3)
      Alpha3=table(4)
      Alpha4=table(5)
      Alpha5=table(6)
      Alpha6=table(7)

      KBeta=table(8)
      Beta1=table(9)
      Beta2=table(10)
      Beta3=table(11)
      Beta4=table(12)
      Beta5=table(13)
      Beta6=table(14)
      AlphaModulus=table(15)
      BetaModulus=table(16)

      temp=table(18)
      NorminalStrainrate=table(19)
      NewYieldStress=table(20)
```

```
        A=58.23d0
        B=3.54d0
        m=0.777d0
        Eref=0.001d-3
        tr=293.d0
        tm=553.d0

        OldYieldStress=(A+B*log10(NorminalStrainrate/Eref))*
     1                (1-((temp-tr)/(tm-tr))**m)
C
        DeltaStrain= abs(strainNew-strainOld)
        StrainRate = DeltaStrain/dt
        StrainRate = max(StrainRate,1.d-10)
        StrainRate = min(StrainRate,1.d4)
        if(strainNew==0) then
          syield=0
          HAlpha1=0
          HAlpha2=KAlpha*Alpha3*(StrainRate**Alpha5)*exp(Alpha6/temp)
          HBeta1=0
          HBeta2=KBeta*Beta3*(StrainRate**Beta5)*exp(Beta6/temp)

hard=AlphaModulus/(AlphaModulus+BetaModulus)*(HAlpha1+HAlpha2)
     1     +BetaModulus/(AlphaModulus+BetaModulus)*(HBeta1+HBeta2)
          hard=hard*NewYieldStress/OldYieldStress
        else
          p1=exp(-Alpha1*strainNew)+strainNew**Alpha2
          p2=1-exp(-Alpha3*strainNew)
          p3=exp(Alpha4*strainNew)*exp(Alpha6/temp);

          p4=exp(-Beta1*strainNew)+strainNew**Beta2
          p5=1-exp(-Beta3*strainNew)
          p6=exp(Beta4*strainNew)*exp(Beta6/temp);

          HAlpha1=KAlpha*p1*p2*p3*Alpha5*(StrainRate**(Alpha5-1))/dt
          HBeta1=KBeta*p4*p5*p6*Beta5*(StrainRate**(Beta5-1))/dt

          HAlpha2=KAlpha*p3*(StrainRate**Alpha5)*
     1    ( (-Alpha1+Alpha4)*exp(-Alpha1*strainNew)
     2      + (Alpha1+Alpha3-Alpha4)*exp(-(Alpha1+Alpha3)*strainNew)
     3      + (Alpha2+Alpha4*strainNew)*(strainNew**(Alpha2-1))
     4      + (Alpha3*strainNew-Alpha2-Alpha4*strainNew)*
```

```
     5          (strainNew**(Alpha2-1)*exp(-Alpha3*strainNew))
     6    )


      HBeta2=KBeta*p6*(StrainRate**Beta5)*
     1 ( (-Beta1+Beta4)*exp(-Beta1*strainNew)
     2      + (Beta1+Beta3-Beta4)*exp(-(Beta1+Beta3)*strainNew)
     3      + (Beta2+Beta4*strainNew)*(strainNew**(Beta2-1))
     4      + (Beta3*strainNew-Beta2-Beta4*strainNew)*
     5          (strainNew**(Beta2-1)*exp(-Beta3*strainNew))
     6    )


C       calcular current yield stress and hardening rate
C

c
hard=AlphaModulus/(AlphaModulus+BetaModulus)*(HAlpha1+HAlpha2)
c     1      +BetaModulus/(AlphaModulus+BetaModulus)*(HBeta1+HBeta2)


hard=AlphaModulus/(AlphaModulus+BetaModulus)*(HAlpha1+HAlpha2)

      hard=hard*NewYieldStress/OldYieldStress

      syieldAlpha=KAlpha*p1*p2*p3*(StrainRate**Alpha5)
      syieldBeta=KBeta*p4*p5*p6*(StrainRate**Beta5)

C        syield =AlphaModulus/(AlphaModulus+BetaModulus)*syieldAlpha
C     1           +BetaModulus/(AlphaModulus+BetaModulus)*syieldBeta

      syield =AlphaModulus/(AlphaModulus+BetaModulus)*syieldAlpha

      !syield = min(syield,200.0)
      syield = syield*NewYieldStress/OldYieldStress
      syield = max(syield,1.d-5)
    endif
    end
```
